# Feasibility of the Aktivplan Digital Health Intervention for Supporting Regular Physical Activity Following Phase II Rehabilitation: Randomized Controlled Pilot Feasibility Study (ACTIVE-CaRe Pilot)

**DOI:** 10.2196/90203

**Published:** 2026-08-11

**Authors:** Dirk Leysen, Daniela Wurhofer, Isabel Höppchen, Eva Maria Bamberger, Aliz Breda, Anna Eleonora Carrozzo, Rik Crutzen, Robert Danner, Ioana Duca, Vincent Grote, Devender Kumar, Veronika Leitner, Barbara Mayr, Hannah McGowan, Josef Niebauer, Franziska Pfannerstill, Bernhard Reich, Mahdi Sareban, Jan David Smeddinck, Gunnar Treff, Stefan Tino Kulnik

**Affiliations:** 1 Ludwig Boltzmann Institute for Digital Health and Prevention, Salzburg, Austria; 2 Care and Public Health Research Institute, Department of Health Promotion, Maastricht University, Maastricht, Limburg, The Netherlands; 3 Salzburg Research Forschungsgesellschaft mbH, Jakob-Haringer-Straße 5/3, Salzburg, 5020, Austria, 43 6622288200; 4 Reha-Klinik Montafon, Schruns, Austria; 5 REHA Zentrum am Uniklinikum Salzburg, Salzburg, Austria; 6 Ludwig Boltzmann Institute for Rehabilitation Research, Vienna, Austria; 7 Center for Industrial Software, University of Southern Denmark, Odense, Denmark; 8 University Institute of Sports Medicine, Prevention and Rehabilitation, Salzburg, Austria; 9 Institute of Molecular Sports and Rehabilitation Medicine, Paracelsus Medical Private University, Salzburg, Austria

**Keywords:** behavior change, cardiac rehabilitation, cardiovascular disease, digital technology, eHealth, exercise, habit formation, mobile health, secondary prevention, smartphone app

## Abstract

**Background:**

Patients with cardiovascular disease (CVD) often struggle to develop and maintain heart-healthy physical activity habits, even after completing a cardiac rehabilitation program. Digital tools offer opportunities to support long-term behavior change in secondary prevention. The aktivplan digital health intervention (DHI) was designed to help patients establish heart-healthy physical activity routines.

**Objective:**

This study aimed to evaluate the feasibility of a randomized controlled trial design to assess the effectiveness of the aktivplan DHI for patients with CVD or increased CVD risk.

**Methods:**

This was a pilot feasibility study with a 2-arm, parallel-group, nonblinded randomized controlled design. Patients admitted to 2 phase II rehabilitation centers in Austria were screened. Eligible participants were patients admitted for cardiac rehabilitation and those admitted for noncardiac rehabilitation who had an increased cardiovascular risk. Recruited patients were randomly assigned to the aktivplan intervention or standard care without digital support. Before discharge, the intervention group was given access to the aktivplan app and a personalized prospective physical activity plan. Data collected at baseline, discharge from the rehabilitation center, and 10-week follow-up included clinical assessments, patient-reported outcomes, and wearables (accelerometry, heart rate). All patients participated in qualitative interviews. Data on intervention implementation were gathered from all health care professionals (HCPs) through questionnaires and a focus group. The qualitative data analysis was conducted according to the framework analysis method. Feasibility outcomes included recruitment rate, attrition, data completeness, adverse events, patient adherence to the aktivplan intervention, fidelity of intervention delivery regarding shared decision-making by HCPs, and both patients’ and HCPs’ experiences of the intervention and study procedures. All analyses were descriptive.

**Results:**

From October 2023 to August 2024, a total of 34 participants (men: n=21, 62%, mean age 57, SD 14 y) were recruited. Sixteen (47%) participants were allocated to the intervention group, and 18 (53%) to the control group. At study sites 1 and 2, the recruitment rate was 1.0 and 0.6 patients per week, respectively. Randomization resulted in equal groups with respect to sex and physical fitness. Attrition was 18%, and data completeness was 97.4%. There were no adverse events related to the intervention or study procedures. Patient adherence was high, with 71% of planned physical activities completed and a mean of 39 (SD 38) additional physical activities per participant. Participants and HCPs were generally supportive of the intervention and the study procedures, providing positive feedback and helpful suggestions for the improvement of design and intervention. HCPs felt that the intervention was implemented effectively (Normalization Measure Development Questionnaire: mean score 3.75, SD 0.50; scale 1‐5, with a higher score indicating better implementation).

**Conclusions:**

This pilot study generated data to inform the design of a future definitive effectiveness trial of the aktivplan DHI, including strategies for optimizing recruitment and the acceptability of the intervention.

## Introduction

### Background

Cardiovascular disease (CVD) remains the leading cause of death worldwide, with 18.6 million deaths attributed to CVD in 2019 [1]. In Austria, CVD accounted for 34.7% of all deaths in 2023 and contributed to 11.7% of early retirements [2]. Cardiac rehabilitation programs are a vital component of the secondary prevention of CVD [3,4]. These programs aim to assist patients in adopting heart-healthy habits, including regular physical activity, through supervised sessions, self-directed training at home, or a combination of both [3].

Cardiac rehabilitation is effective in reducing long-term morbidity and mortality and is consistently endorsed in contemporary clinical guidelines for conditions such as chronic heart failure, post-myocardial infarction, and chronic coronary syndromes [5]. However, completing a cardiac rehabilitation program does not always lead to long-term adherence to physical activity [6]. This challenge in maintaining heart-healthy physical activity after rehabilitation has been recognized by medical societies. In 2019, the European Research Area Network for Cardiovascular Diseases identified this issue as a research priority, emphasizing the need for strategies to support lifestyle maintenance, potentially through digital technologies [7]. The European Society of Cardiology’s guidelines also highlight the gap in long-term adherence to physical activity and suggest that eHealth tools could help address this challenge [8].

Digital technologies offer potential innovative solutions for long-term and sustainable habit formation for independent, regular, heart-healthy exercise. In a systematic review [9] that included 31 studies, the authors concluded that digital technologies have the potential to enhance care and broaden access to cardiac rehabilitation through tailored interactive interventions. Furthermore, other reviews [10,11] indicate that supporting physical activity through digital technologies following a cardiac rehabilitation program can achieve higher physical activity and physical performance outcomes compared to standard care without digital technology support following a cardiac rehabilitation program. Nevertheless, this approach remains an ongoing area of investigation. In the European region, for example, the “CoroPrevention” [12] and “INTERCEPT” [13] studies investigate technology-supported comprehensive secondary prevention for patients with CVD, whereby these intervention concepts address all cardiovascular risk factors rather than focusing on physical activity alone. In this context, this study explored the feasibility of a future randomized clinical trial on the novel digital health intervention (DHI) “aktivplan” [14]. The aktivplan DHI was specifically designed for the Austrian rehabilitation pathway to help patients establish and maintain regular heart-healthy physical activity after completing phase II cardiac rehabilitation. Phase II rehabilitation describes a multidisciplinary rehabilitation program following shortly after the acute care (phase I) [15,16]. Given that health care systems and rehabilitation pathways vary across countries, generating evidence specific to the Austrian context is crucial [17]. On the basis of a recent survey of cardiac rehabilitation professionals, there is no comparable DHI currently established in routine clinical practice in Austria [18]. Importantly, digital tools available from the sports and leisure industry lack the rigorous scientific investigation and clinical effectiveness evidence necessary for implementation in a medical context. Beyond its national relevance, the feasibility insights gained in this study may also inform the design and implementation of future mobile health–based randomized controlled trials (RCTs) in comparable international health care and rehabilitation settings.

### Study Aim

The aim of this study was to assess the feasibility of a future RCT of the aktivplan DHI. Quantitative feasibility measures included recruitment rate, attrition, data completeness, adverse event reporting, patient adherence to the aktivplan intervention, and fidelity of intervention delivery with respect to shared decision-making (SDM) by the health care professionals (HCPs). Qualitative feasibility data addressed participants’ use of alternative and additional strategies for supporting physical activity, HCPs’ reflections on SDM fidelity, and both participants’ and HCPs’ experiences and perceptions of the intervention and study procedures.

## Methods

### Study Design

This was a pilot feasibility study with a 2-arm, parallel-group, nonblinded RCT design. Both quantitative and qualitative data collection methods were used. The study was conducted at 2 rehabilitation centers in Austria. A protocol paper has been published [19]. The reporting of this study adheres to the CONSORT (Consolidated Standards of Reporting Trials) statement extension for randomized pilot and feasibility trials [20] and to the TIDieR (Template for Intervention Description and Replication) checklist [21]. The CONSORT and TIDieR checklists are provided as Checklist 1 and Checklist 2.

### Ethical Considerations

The study received ethical approval from the research ethics committees of the Austrian federal states of Salzburg (reference 1065/2023) and Vorarlberg (reference EK-2-14/2023) and was registered with the Austrian Federal Office for Safety in Health Care (102214904). Participation in this study was voluntary, and all participants gave written informed consent to take part in the study.

### Setting

The first study site (REHA Zentrum am Uniklinikum Salzburg, Salzburg, Austria; S1) was an outpatient rehabilitation center in an urban setting. Patients attended the center during the day for their rehabilitation appointments, allowing them to work while undergoing rehabilitation. The second study site (Reha-Klinik Montafon, Schruns, Austria; S2) was an inpatient cardiac rehabilitation clinic in a rural location, where patients spent 3 to 4 weeks for their rehabilitation.

### Participants

Eligible participants were adult patients enrolled in the phase II cardiac rehabilitation program at S1 or S2 or undergoing phase II rehabilitation for a noncardiac condition but with increased cardiovascular risk at S1. This increased risk was defined by physical inactivity (<30 min of moderate-intensity exercise on 5 d/wk or <20 min of vigorous-intensity exercise on 3 d/wk), combined with at least one additional cardiovascular risk factor, such as smoking, hyperlipidemia, arterial hypertension, diabetes, or obesity. Detailed inclusion and exclusion criteria are described in the protocol paper [19].

### Recruitment and Randomization

Patients were screened against inclusion and exclusion criteria, as presented in Textbox 1, by site investigators, and all eligible patients were invited to participate in the study. Patients who expressed interest in participating in the study were asked to provide written informed consent. Random allocation was concealed from the recruiting sites and administered by the sponsor organization (Ludwig Boltzmann Gesellschaft) at a different location (Ludwig Boltzmann Institute for Digital Health and Prevention, Salzburg, Austria). Randomization was stratified by study site, sex, and exercise capacity (peak power per body weight in maximal cycle ergometry at admission to rehabilitation).

Textbox 1. **Inclusion and exclusion criteria.**

Inclusion criteria

- Adult patients enrolled in either a phase II cardiac rehabilitation program or phase II rehabilitation due to a noncardiac indication but who present with increased cardiovascular risk (ie, physical inactivity with at least one of the following cardiovascular disease risk factors: smoking, hyperlipidemia, arterial hypertension, diabetes, or obesity).
- Possession and use of a smartphone compatible with the aktivplan DHI (Android version 4.4 [Google LLC] or iOS version 11.0 [Apple Inc.] or above and internet access).

Exclusion criteria

- Existent use of a digital intervention to support regular heart-healthy physical activity by the patient already established in standard care at the time of recruitment.
- Medical contraindications to symptom-limited incremental cycle ergometry; regular heart-healthy physical activity, exercise, and sports; or the use of a smartphone.
- Insufficient cardiovascular exercise capacity (eg, due to severe impairment of the musculoskeletal system).
- Participation of the patient in another clinical trial within the last 6 months.
- Lack of mental capacity to consent to study participation (eg, addiction or other illnesses that do not allow the person to assess the nature, scope, and possible consequences of participation in the study).
- Pregnancy in the third trimester or pregnancy in the first and second trimesters with a medical contraindication to physical activity, exercise, and sports.
- Breastfeeding women with a medical contraindication to physical activity, exercise, and sports.
- Indications that the patient is unlikely to comply with the protocol (eg, unable to commit to attending follow-up appointments).

### Intervention

**Figure 1.**
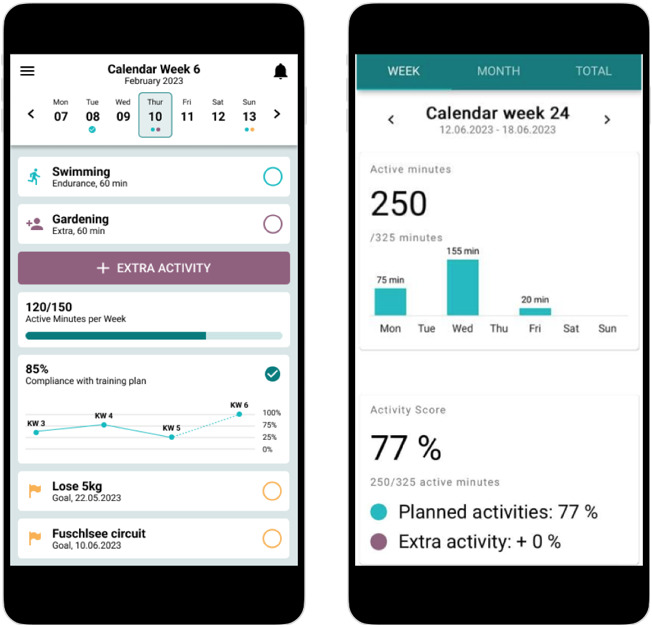
Screenshots of the aktivplan smartphone app for patients. The physical activity plan is developed jointly between the patient and the health care professional using shared decision-making and a person-centered approach. Left: planned physical activity on the day, achieved physical activity of the current week, visualization of physical activity adherence during the preceding 4 weeks, and personal goals. Right: visualization of achieved physical activity and activity score of the current week.

The study intervention began shortly before discharge from the phase II rehabilitation program. Participants allocated to the intervention group were introduced to the aktivplan app (Figure 1) in a one-to-one consultation with an HCP experienced in exercise prescription (a physiotherapist or sports scientist). The patient and HCP discussed and agreed on a personalized heart-healthy physical activity plan to be followed independently for 10 weeks after discharge. This consultation adhered to SDM principles [22]. A detailed description of the intervention is provided in the TIDieR checklist (Checklist 1).

### Control Condition

The control group received standard care according to the Austrian national rehabilitation plan [23,24], which did not include any DHIs during or after phase II rehabilitation. No placebo or alternative comparative treatment was provided.

### Data Collection Procedures

**Figure 2.**
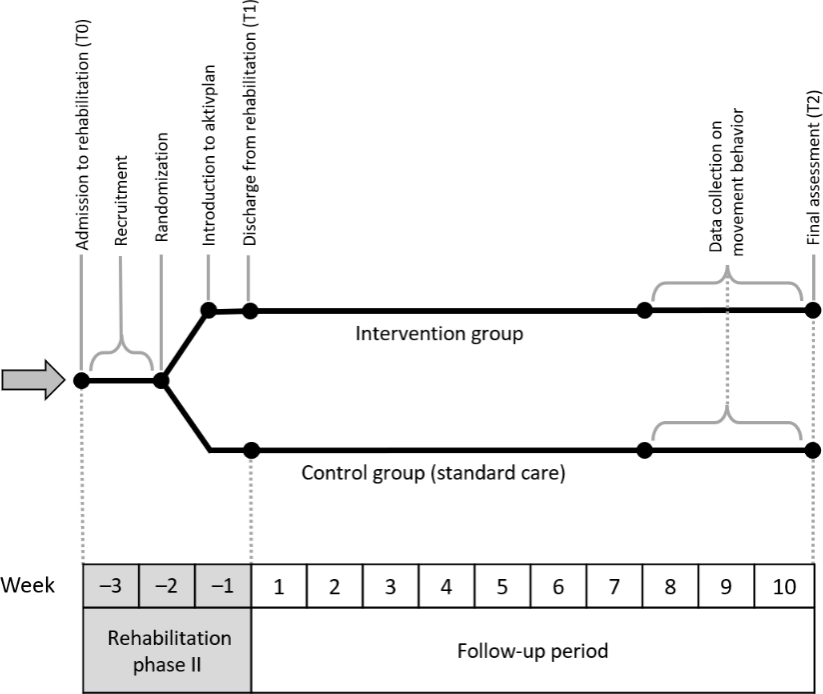
Data collection timeline.

Quantitative data were collected from all participants at 3 time points: baseline (T0), discharge from rehabilitation (T1), and the 10-week follow-up (T2). This included clinical assessments, patient-reported outcome measures, accelerometry, and wearable heart rate sensors. These data are not presented in this paper, but these data collection procedures were assessed with respect to their feasibility and acceptability for patients and HCPs. Figure 2 presents the data collection timeline for patients in this study.

Quantitative data collection from HCPs throughout the study period included the following standardized questionnaires, available in validated German versions. The 9-item Shared Decision Making Questionnaire (SDM-Q-Doc) [25], with a scale from 0 to 45 (higher values indicating a higher level of SDM), was completed by HCPs after each aktivplan physical activity planning session (consultation). The questionnaire was selected to inform about HCPs’ subjective perception of the implementation of SDM principles during the aktivplan consultations. Video recordings of all aktivplan consultations were rated by 2 independent raters using the Observer OPTION^5^ rating scale [26], with a maximum score of 20 points (higher ratings indicating a higher level of SDM). This questionnaire was selected to add an objective rating of accordance with SDM principles, complementing the SDM-Q-Doc. The German Normalization Measure Development Questionnaire [27], with a scale from 1 to 5 (higher scores indicating better intervention implementation), is a 13-item clinician-rated implementation measure that indicates how well a new intervention is implemented in clinical practice. It was completed at the end of the study.

Qualitative data were collected from all participating patients through semistructured interviews, following a qualitative interview schedule, at T2 by members of the study team (DL, IH, and STK). Two were experienced qualitative interviewers (IH and STK), and one was a novice (DL). Before the interviews began, the interview schedule was discussed and piloted with the patient representative on the study team (VL). The first interview of each study team member was observed by a second interviewer and then debriefed. These measures were taken to ensure consistency across the interviewers. Interviews lasted up to 20 minutes and aimed to provide an in-depth understanding of patients’ lived experiences regarding the study procedures and intervention. Qualitative data from all HCPs were collected during and at the end of the study period. Handwritten notes and open responses in questionnaires were gathered throughout the study period. A 2-hour-long focus group discussion was conducted at the end of the study period, moderated by one member of the study team (EB). The aim of the focus group was to facilitate HCPs’ retrospective discussion and reflection on the study procedures and intervention, as well as on other observations regarding the conduct of the study that would need to be considered in the design of a larger definitive trial.

The qualitative interview schedule and the topic guide for the focus group are attached as online supplements 3 and 4 in Multimedia Appendices 1 and 2.

### Feasibility Outcomes

The feasibility of conducting a larger RCT with a similar design was assessed through quantitative measures and qualitative data. Quantitative measures included recruitment rate, attrition, data completeness, adverse event reporting, patient adherence to the aktivplan intervention, and fidelity of intervention delivery with respect to SDM by the HCP. Qualitative data addressed participants’ use of alternative and additional strategies for supporting physical activity, HCPs’ reflections on SDM fidelity, and both participants’ and HCPs’ experiences and perceptions of the intervention and study procedures. There were no prespecified criteria to judge whether, or how, to proceed with a future definitive trial [20]. As no comparable previous studies are available for Austria, and prespecified criteria are not imposed by the funding organization, the decision whether, or how, to proceed with a future definitive trial will be made in discussion with relevant stakeholders and based on the data from this feasibility study and comparable international studies.

### Data Analysis

The statistical data analysis was conducted using Excel for Microsoft 365 (Microsoft Corporation, 2022). For descriptive data analysis, the appropriate summary measures were calculated according to data type, including measures of central tendency and spread for continuous data and frequency, proportions, or percentages for categorical data. Additionally, 95% CIs were calculated for recruitment rates, attrition, data completeness, and adherence data. As this was a pilot study, no confirmatory inferential statistical analysis or hypothesis testing was conducted.

The qualitative data analysis was conducted according to the framework analysis method [28] using QDA Miner Lite (Provalis Research, version 3.0.5). Recordings of the patient interviews and the focus group with HCPs were transcribed verbatim by a professional transcription agency. The transcripts were checked for accuracy against the original recordings by the first author (DL). Afterward, DL familiarized himself with the transcripts by rereading and coding them iteratively using the questions stated in the interview guide and focus group schedule as the basis for the deductive framework. Within each framework category, the interpretation and comparison of these codes among participants led to the definition of analytic themes against the research questions. The presentation of these themes was supported and illustrated with selected anonymized quotes. During this analysis process, DL conducted several peer review meetings with other study team members experienced in qualitative research (IH, RC, and STK) to enhance the consistency and credibility of the analysis. Interpretive decisions were documented in QDA Miner Lite for auditability. In the reporting of qualitative findings, care was taken to preserve the anonymity of the patients and HCPs.

## Results

### Baseline Characteristics

A total of 34 participants, with 16 (47%) allocated to the intervention group and 18 (53%) allocated to the control group, were recruited at both study sites, including 20 (59%) at S1 and 14 (41%) at S2. Participants’ mean age was 57 (SD 14) years, 62% (n=21) were men, and the mean peak power at baseline was 1.62 (SD 0.47) W/kg. Randomization resulted in balanced groups between intervention and control regarding sex and exercise capacity. The intervention group was, on average, 9 years younger. The characteristics of the sample according to study group are presented in Table 1. Approximately half (18/34, 53%) of the participants had never smoked or were ex-smokers. Prior to the start of rehabilitation, only half (17/33, 43%) of the participants reported being physically active according to the Rapid Assessment of Physical Activity I questionnaire, and a third (12/33, 36%) reported engaging in muscle strength and/or flexibility exercises on the Rapid Assessment of Physical Activity II questionnaire [29]. All participants reported having used a smartphone several times per day prior to their admission to rehabilitation, and one quarter (8/33, 24%) reported having used a wearable device at least once per day prior to their admission to rehabilitation.

**Table 1.** Baseline characteristics of the sample (N=34).

Characteristics	Control (n=18)	Intervention (n=16)
Study site, n (%)		
Site 1	9 (50)	11 (69)
Site 2	9 (50)	5 (31)
Sex, n (%)		
Male	11 (61)	10 (63)
Female	7 (39)	6 (37)
Peak power (W/kg), mean (SD)	1.64 (0.46)	1.6 (0.49)
Age (y), mean (SD)	61.6 (11.8)	52.3 (15.4)
Height (cm), mean (SD)	173.7 (12)	175.4 (11.5)
Weight (kg), mean (SD)	88.9 (20.5)	98.1 (24.7)
Smoking, n (%)		
No	9 (50)	4 (25)
Yes	7 (39)	9 (56)
Ex-smoker	2 (11)	3 (19)
Marital status, n (%)^a^		
Married or in a registered partnership	13 (72)	12 (80)
Single	1 (6)	3 (20)
Divorced	4 (22)	0 (0)
Employment status, n (%)		
Employed	8 (44)	10 (63)
Unemployed	1 (6)	0 (0)
Retired	9 (50)	5 (31)
Student	0 (0)	1 (6)
Rehabilitation phase III^b^, n (%)^c^		
Not informed	6 (40)	5 (42)
Informed	3 (20)	3 (25)
Referred	2 (13)	2 (17)
Not interested	4 (27)	2 (17)
RAPA I, n (%)^a^		
Sedentary	0 (0)	1 (7)
Underactive	5 (28)	1 (7)
Underactive regular–light activities	—	—
Underactive regular	4 (22)	5 (33)
Active	9 (50)	8 (53)
RAPA II, n (%)^a^		
None	10 (56)	11 (73)
Muscle strengthening, once a week or more	5 (28)	3 (20)
Flexibility exercises, once a week or more	0 (0)	0 (0)
Both	3 (17)	1 (7)
Smartphone use, n (%)^a^		
Multiple times a day	18 (100)	15 (100)
Wearable use, n (%)^a^		
Several times a day	2 (11)	2 (13)
Once a day	3 (17)	1 (7)
4‐6 d/wk	1 (6)	1 (7)
1‐3 d/wk	0 (0)	0 (0)
Less than once a week	2 (11)	1 (7)
Never	7 (39)	8 (53)
Don’t know	3 (17)	1 (7)

a Participants in the control group (n=18) and participants in the intervention group (n=15).

b Following completion of phase II rehabilitation, patients in Austria are eligible to apply for phase III rehabilitation, which is an outpatient program with approximately weekly sessions over 6 to 12 months.

c Participants in the control group (n=15) and participants in the intervention group (n=12).

### Recruitment Rate

**Figure 3.**
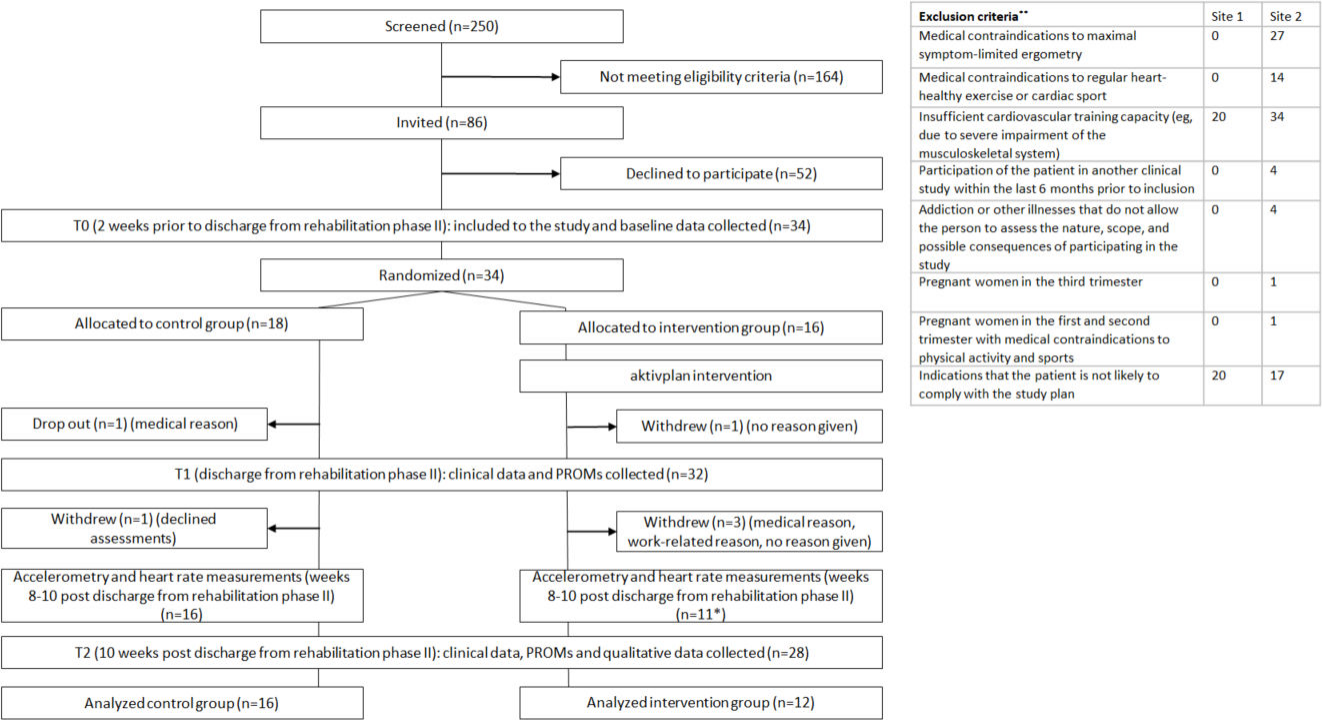
CONSORT (Consolidated Standards of Reporting Trials) flow diagram. *One participant did not complete accelerometry and heart rate measurements but did complete T2 assessments. **Multiple exclusion criteria may apply to a single excluded participant. PROM: patient-reported outcome measure.

The CONSORT flow diagram is presented in Figure 3, along with the reasons for exclusion at each study site. The recruitment period at S1 lasted 20 weeks, from October 2023 to February 2024. Of 123 patients screened, 54 were eligible and invited, accounting for 44% (95% CI 35-53) of the screened patients, and 20 were recruited, resulting in a proportion of 16% (95% CI 10-23) of the screened patients and 37% (95% CI 24-50) of those invited. The average recruitment rate at S1 was therefore 1.0 (95% CI 0.56-1.44) patient per week. One participant at S1 underwent cardiac rehabilitation. All other participants at S1 underwent rehabilitation for noncardiac indications and had insufficient physical activity with one or more additional risk factors for CVD. The most common additional risk factors were hypercholesterolemia (n=16), hypertension (n=12), and obesity (n=11).

At S2, the recruitment period lasted 24 weeks from October 2023 to April 2024. Of 127 patients screened, 32 were eligible and invited, accounting for 25% (95% CI 18-33) of the screened patients, and 14 were recruited, corresponding to 11% (95% CI 6-16) of the screened patients and a proportion of 44% (95% CI 27-61) of the invited patients. The average recruitment rate at S2 was 0.6 (95% CI 0.28-0.89) patients per week, approximately 1 participant every 2 weeks.

In total, 164 participants did not meet the eligibility criteria for participation. At S1, the 2 most common exclusion criteria were inability to comply with exercise training recommendations (n=20, 12%), mostly due to pain, functional impairments, and recent knee or hip surgery; and likely noncompliance with the study plan (n=20, 12%) mostly due to depression, language barriers, and psychological or social circumstances. At S2, the most common exclusion criteria were inability to comply with exercise training recommendations (eg, due to severe musculoskeletal impairment, n=34, 21%), as well as medical contraindications to maximal symptom-limited ergometry (n=27, 16%). Among the latter, blood pressure limitations caused by aortic dissection, ectasia, or aneurysm were the most prevalent. In addition, likely noncompliance with the study plan (n=17, 10%) was observed primarily due to language barriers or lack of willingness to cooperate.

### Attrition

A total of 6 participants withdrew during the study, representing 18% (95% CI 5-30) of all 34 participants. Comparing the 2 groups, 25% (n=4, 95% CI 7.0-41.0) withdrew from the intervention group, and 11% (n=2, 95% CI 0-23) withdrew from the control group. Comparing the gender, 4 (66%) of 6 were men, of whom 3 (50%) were in the intervention group and 1 (17%) was in the control group. The 2 (33%) female participants who withdrew were 1 in the intervention group and 1 in the control group. Comparing the 2 study sites, 16 (80%) of 20 participants completed their final study visit (T2) at S1 (outpatient center), resulting in 20% (n=4, 95% CI 0-40) attrition, with all 4 (100%) participants who withdrew allocated to the intervention group. At S2 (inpatient center), 12 (86%) of 14 participants completed T2, resulting in 14% (n=2, 95% CI 0-33) attrition, with both participants who withdrew allocated to the control group.

### Data Completeness

The case report form specified a total of 8423 data entries for 34 patients across all 3 data collection points (T0, T1, and T2). The number of missing data entries was 217 (data completeness 97.4%, 95% CI 97.1 to 97.8).

### Adverse Events

During the 10-week study period, a total of 15 (44%) participants reported 17 adverse events (control group: n=7, 47% and intervention group: n=8, 53%; men: n=11, 73% and women: n=4, 27%). Of these events, one in the control group was classified as a serious unexpected event. This patient experienced a cerebral hemorrhage, which was not related to the study. The remaining 16 adverse events were classified as unexpected events, including respiratory infection, diarrhea, musculoskeletal injury, hypertension, and others. Seven unexpected events occurred in the control group, and 9 occurred in the intervention group. None were related to the intervention or to other study procedures. Of the 15 participants experiencing adverse events, 2 (13%) withdrew from the study.

### Adherence to the Aktivplan Intervention

Throughout the intervention period, participants in the intervention group manually confirmed completion of their planned physical activity sessions and could also enter additional (unplanned) physical activity sessions in the aktivplan app. Among the 12 participants (7 men and 5 women) in the intervention group who completed T2, the mean number of planned physical activity sessions over the 10-week period was 86 (SD 33; men: 79, SD 33, and women: 97, SD 33). The overall completion rate was 70% (n= 61, 95% CI 61-80; men: 72%, n=57, 95% CI 62-82; and women: 69%, n=67, 95% CI 59-78). Besides these planned sessions, participants performed on average 39 (SD 38) additional (unplanned) physical activity sessions (men: 51, SD 43, and women: 21, SD 23), amounting to 45% (95% CI 34-55) of their originally planned sessions (men: 65%, n=51, 95% CI 55-76 and women: 22%, n=21, 95% CI 13-30). Overall, during the 10-week intervention, all participants completed on average 100 (SD 53) physical activity sessions (men: 108, SD 54, and women: 88, SD 56), combining both planned and additional (unplanned) sessions.

### Use of Alternative and Additional Strategies for Supporting Physical Activity

Among the participants in the intervention group, 4 (33%) participants reported alternative and additional strategies for supporting physical activity during the study period, including digital technology (eg, other smartphone apps, wearable devices, and online exercise instructions; n=2, 17%), one-to-one support (eg, personal trainer or physiotherapist; n=1, 8%), enrollment in regular supervised exercise classes and fitness center memberships (n=3, 25%), and enrollment in phase III rehabilitation (n=1, 8%). In the control group, 9 (56%) participants reported alternative and additional strategies for supporting physical activity, including digital technology (n=2, 12%), one-to-one support (n=3, 19%), enrollment in regular supervised exercise classes and fitness center memberships (n=5, 31%), and enrollment in phase III rehabilitation (n=1, 6%).

### Implementation of the Aktivplan Intervention

Four HCPs (3 women and 1 man; 3 physiotherapists and 1 sports scientist) delivered the aktivplan DHI at the study sites, with 2 HCPs per site. Prior to the start of recruitment, all HCPs attended a 1-day training event focused on implementing the aktivplan DHI in the context of the study.

The implementation of the study intervention by HCPs was evaluated using the German Normalization Measure Development Questionnaire at the end of the study. HCPs reported a mean score of 3.75 (SD 0.50). In the subcategories, the HCPs scored a mean of 3.67 (SD 0.86) for coherence, 3.81 (SD 0.80) for cognitive participation, and 3.25 (SD 0.32) for collective action.

In the focus group discussion, HCPs reported that, after the training event, they had felt well prepared to deliver the study intervention. However, because the training events took place several months before the first patient was recruited, some HCPs felt somewhat uncertain when the time came to deliver the intervention for the first time. One HCP suggested that a short refresher training just before the first actual patient session would have been helpful:

*If it’s been a while, just a quick refresher. And you could do that via Zoom, for example, saying, “Hey, come on, let’s go through it again quickly.” It was good, but it was a lot of information for one day.*

[HCP1]

### Patients’ and HCPs’ Experiences Regarding the Intervention

In qualitative interviews, patients provided overall positive feedback on the aktivplan intervention and the app, stating that “I think the app is great” (patient A, female, 37 y), “everything went well” (patient B, male, 31 y), and “everything is fine for me” (patient C, male, 67 y). When asked about the influence of the app on their physical activity during the intervention period (“Using aktivplan has affected my activity level as follows”), 7 (58%) answered “much more active,” 2 (17%) answered “more active,” 3 (25%) answered “unchanged,” and none of the patients answered “less” or “much less.” With regard to participants’ intention to train (“I plan to continue regular weekly training after completing rehabilitation”), the trend from T0 to T1 and T2 in the intervention group showed mean scores of 6.53 (SD 0.92), 6.40 (SD 1.55), and 6.58 (SD 0.90), respectively, on a scale from 1 to 7 (higher values indicating greater intention). The control group had mean scores of 6.50 (SD 1.2), 5.94 (SD 1.77), and 6.19 (SD 1.47), respectively.

In the focus group discussion, HCPs reported that they had sometimes experienced time pressures during the one-to-one consultation due to restricted time schedules, language barriers, or minor technical issues, such as poor internet connection. For example, one HCP explained:

*One patient took longer because he didn't speak German very well. So, we had to do a bit of translating, or try to explain things in very slow and understandable sentences. That made it a bit more difficult. It took longer than usual.*

[HCP1]

During this one-to-one consultation between the HCP and their patient, a prospective physical activity plan tailored to the patient’s needs was scheduled in the aktivplan calendar. Compared to their usual practice, this enabled the HCPs to develop a more consistent, structured, and personalized plan together with their patients. This instilled confidence in the intervention, as reported by one of the HCPs when asked what they did differently when using aktivplan:

*Otherwise, I always say, it would be good to do something for 15 or 20 minutes every day. And that [aktivplan physical activity planning] was just much more precise. Much more precisely planned, much more precisely structured exercises, so to speak.*

[HCP1]

While 3 HCPs found that the app was generally well designed and overall successful, 1 HCP was particularly critical of the app. One major criticism concerned the number of steps required to schedule and log personalized physical activities, which highlights an area for future development, for example, by increasing automation without reducing the individual touch of the planning:

*It’s really just a calendar app. And I think there are many better sports apps out there that are easier for people to use, because you have to enter every single activity you do manually [in the aktivplan app]. I think that’s quite a lot of effort. There are a few people who need something like this, who enjoy doing it. For them, maybe, if it’s a bit better developed, I'd say. But basically, I don’t really see the point, especially in the long term.*

[HCP3]

Throughout the entire study period, the HCPs monitored their allocated patients’ physical activity adherence using the aktivplan web application. HCPs reported that this monitoring task was smoothly integrated into their daily routines. However, they suggested that the application lacked features that could have facilitated 2-way communication. For example, an easy way for patients to confirm whether the agreed physical activity plan was suitable and effective would be helpful, as one HCP remarked:

*It [offering support after discharge from rehabilitation] was quite difficult, because I didn’t receive any feedback at all.*

[HCP3]

A second observation related to communication was that patients used the note-writing feature in the aktivplan app in various ways. When marking a physical activity as completed in the app, patients could annotate additional information related to this physical activity. Some patients did not use this function or made notes for themselves, while others wanted to communicate with their HCP or place questions to their HCP in these notes. As these notes did not trigger any notifications for the HCP, they remained unnoticed, or HCPs had to manually review each patient’s physical activity entries to spot new notifications. This was not always possible and created an additional burden on the HCPs:

*There’s no notification of anything, and you have to click through every activity to see if the participant has written something.*

[HCP3]

In addition to creating an increased workload for HCPs, these communication issues sometimes also caused misunderstandings and perceived lack of support for patients, as shown by 1 participant’s notes in the app:

*Isn’t Tuesday’s rehab enough for you, and yesterday’s 1 hour walk??*

[Patient D, male, 38 years, dropout]

A few days later, the same patient wrote:

*Since the study is voluntary, I reserve the right to terminate my participation because I am no longer interested in playing the guinea pig.*

[Patient D]

Another observation that seemed to create some disappointment for patients was that the graph that visualizes adherence to the planned physical activities did not include additional unplanned physical activities:

*What often happened was that patients didn't do the planned activities, but did something else instead. Then they still only got 80% or something, even though they actually exceeded the minutes and what they did. That didn't really add up.*

[HCP1]

A related issue raised by patients was that the app did not allow rescheduling of planned activities to different days, which would have been helpful:

*Yes, definitely incorporate more flexibility for the user so that training sessions can be rescheduled more easily.*

[Patient E, female, 28 years]

In summary, while patients’ and HCPs’ overall feedback on the aktivplan intervention was positive, the qualitative findings identified several areas for improvement of the app, including better two-way communication between patient and HCPs, visualization of completed unplanned physical activity sessions in the app, and the ability for patients to reschedule planned physical activity sessions.

### Implementation of SDM in Physical Activity Planning

As part of the aktivplan consultation session, HCPs were instructed to implement SDM when discussing the personalized physical activity plan with the patient. Two independent raters using the Observer OPTION^5^ scale produced comparable results with mean scores of 16.1 (SD 3.4) and 16.1 (SD 4.32). The SDM-Q-Doc showed an increase in total scores with successive consultations. The mean score for the first conversation was 25.25 (SD 5.56), and it increased to 27.27 (SD 3.59) after the second SDM conversation. Three HCPs completed the questionnaire a third time, with a mean score of 28.67 (SD 5.51). Two HCPs participated in the 4th and 5th interviews, with respective mean scores of 32.00 (SD 1.41) and 33.50 (SD 0.71). Patients were asked to reflect on their experience of the aktivplan consultation session and reported positive experiences with their interactions with the HCPs, mostly describing them as “interesting” or “good.” Patients did not specifically mention recognizing SDM as a particular aspect of their HCPs’ communication.

During the focus group, the HCPs were asked about their experience of incorporating SDM. The HCPs reported that the introduction workshop was well conducted and that they appreciated having the theory and history of SDM explained. In particular, they found the practical role-play valuable, in which they acted out the aktivplan consultation session with each other, taking on the roles of the patient, the HCP, and the observer using the Observer OPTION^5^ scale:

*I think he [presenter] spent a lot of time on the theory and explained it in great detail, including shared decision-making. We also did the role-plays. That was very good for getting the background information.*

[HCP3]

HCPs acknowledged the benefits of SDM based on their experience in the study. For example, HCP2 said:

*I would say absolutely. It was crucial for me. Because at first, it was often a bit like, oh God, they [the patients] are given a training plan and they have to go to the gym, for example. So, it was often an “aha’”moment for the patients. Ah, okay, it’s based on mutual discussion and agreement. Okay, that’s cool.*

[HCP2]

### Patients’ Experiences Regarding Study Procedures

Patients’ experience of study procedures was generally good, but some areas for improvement were pointed out. All patients found that they had been sufficiently informed about the study prior to giving consent and that the study information sheet and the explanations given by site investigators had been clear. The most common reason why patients were motivated to participate was to have specific guidance after the phase II rehabilitation program, as 1 participant noted:

*Simply that my work is actually being monitored [by the HCPs], that I can see whether I have improved over time. And also, simply that I persevere with everything. It’s not a bad thing to have a little pressure. And mainly, actually, to encourage my own work a little more.*

[Patient F, female, 73 years, intervention group]

Additionally, interest in science or helping others served as motivators to participate in this study:

*I also want it to be scientifically investigated.*

[Patient G, male, 73 years, control group]

*First and foremost, that I might be able to help other people.*

[Patient H, male, 67 years, intervention group]

In general, undergoing the additional procedures as part of the study did not pose a problem for patients, although some found the questionnaires too long and not always easy to understand:

*In my opinion, they [questionnaires] need to be reworded. For example, pensioners are not considered at all, and it is sometimes very difficult to understand correctly.*

[Patient I, female, 61 years, control group]

The wearable measurement devices (not part of the intervention), worn daily during the last 3 weeks of the study, were also generally well accepted, but some participants found it bothersome to wear 2 devices and to have to charge devices daily. All patients (n=28) completed the Comfort Rating Scale [30], which assesses 6 dimensions (ie, emotion, attachment, harm, perceived change, movement, and anxiety) of wearing a wearable device on a scale from 1 to 10 (with higher values indicating greater discomfort). The mean rating for emotion was 0.93 (SD 1.88), attachment was 2.89 (SD 3.39), harm was 1.61 (SD 2.62), perceived change was 1.21 (SD 2.17), movement was 1.36 (SD 2.08), and anxiety was 0.61 (SD 0.69). The overall mean rating across all dimensions was 1.43 (SD 2.37).

When asked about their thoughts on a 6‐ to 12-month follow-up period, as would be required for a definitive effectiveness study, responses were mixed. Some participants recognized the value of long-term studies, but most felt that a longer study duration would negatively impact recruitment and retention. Approximately half of the participants said they would commit, while the other half said they were unlikely to sign up for a study lasting 6 to 12 months.

## Discussion

### Principal Findings

This pilot study has demonstrated the feasibility of the study design for an RCT of the aktivplan DHI for supporting regular physical activity following phase II rehabilitation. Several factors should be considered before conducting a definitive RCT.

The stratified randomization procedure worked well, resulting in balanced groups. To avoid a significant age difference in both groups, it should be considered to include age as an additional stratification factor in the randomization process.

The average recruitment rate was 1 and 0.6 participants per week for S1 and S2, respectively, and was lower than the expected recruitment rate of 1 participant per week per study site. The difference in recruitment rate might be related to the different settings. The outpatient rehabilitation setting can be particularly attractive to patients who require more flexible rehabilitation provision to accommodate work or family commitments. Patients in outpatient rehabilitation also tend to be more functionally able patients, while the inpatient setting is particularly suitable for patients with greater care needs [31]. These recruitment rates help us estimate and plan the recruitment period and the required number of study sites needed for a future trial. Recruitment rate is a major factor that determines the duration of a study. In digital health, it is noteworthy that the pace of digital technology development generally by far outruns the generation of clinical evidence, as a systematic review of 71 digital intervention studies in CVD, diabetes, mental health, and other health conditions has shown that primary data collection lasted on an average of 6 (SD 8) months [32]. Therefore, it is important to improve the recruitment rate, for example, by increasing the number of study sites, so the intervention stays aligned with the pace of innovation in mobile health.

With an attrition of 18% during this 10-week study, this design appears promising. Comparable studies in cardiac rehabilitation with a 1-year duration have reported attrition rates between 26% and 31% [33-36]. In 9 RCTs with app-based interventions for various chronic diseases, the average attrition was 40% (95% CI 16‐63) for follow-up periods of up to 1 year [37]. On the basis of this, there is a risk of higher attrition in a definitive study with a longer follow-up period than in this pilot study. A systematic review of 52 health behavior trials did not identify any statistically significant influence on attrition of the amount of human contact in the study delivery, the intensity of the intervention and control condition, the type of control condition, follow-up intensity, or follow-up duration. We therefore suggest that it will be important to ensure during the recruitment process that participants’ intention to complete the study is high [38].

The data collected were comprehensive and successful, with data completeness above 95%, which is generally considered successful in health care research [39]. This demonstrates that the guidance, monitoring, and support offered to site investigators were adequate. We recognize the burden of the large number of questionnaires included in this pilot study. This poses the risk of survey fatigue or satisficing [40,41]. However, many of these questionnaires addressed objectives of this pilot feasibility study and will not be required in the future trial.

Considering participant safety, none of the 17 adverse events were considered to be related to the intervention or study procedures, indicating that neither the intervention nor the study design posed a risk to participants. The inclusion and exclusion criteria for this study were rigorous regarding participants’ language skills and physical abilities of the participants but still comparable with those of other studies in this field [34,35]. To improve equity, the inclusion and exclusion criteria should be adapted where possible in the future trial.

Participants (9/12, 75%) in the intervention group reported being “much more” or “more” active due to the aktivplan app. This high activity level is also visible in the adherence to “aktivplan.” Additional unplanned activities cumulatively increased the total number of performed activities above the level of the planned activities. It is important to note that both the control and intervention groups showed similar trends in their intentions to train after rehabilitation, indicating that both groups planned to train regularly after rehabilitation. Interestingly, participants in the control group were more than twice as involved in one or more alternative and additional strategies to support physical activity. This could indicate that “aktivplan” serves as this support in the intervention group. The needs of people with cardiovascular disease for physical activity support and possible opportunities by eHealth were evaluated in a cross-sectional study in 2022 [42]. The positive effect of digital support for physical activity is supported by previous studies [10,11]. It is important to capture the use of alternative and additional physical activity support strategies also in a future definitive trial to evaluate group differences and potential impact on physical activity outcomes.

The patients and HCPs were generally satisfied with the intervention; however, based on qualitative feedback, 3 modifications could enhance the intervention. First, communication between patients and HCPs could be improved by separating notifications—whether personal or intended for HCPs—from questions. An instant alert for incoming messages in the HCP’s web application would enable on-demand response communication. Nevertheless, patients’ communication preferences should be considered, as these will depend on the type of technology user, and some may prefer restricted communication options [43]. Second, personalized physical activity planning could be enhanced by providing more and adaptable exercise-related content within the aktivplan app so that the physical activity planning can be optimally tailored to patient needs [44]. Third, adherence to the aktivplan app could be enhanced by enabling rescheduling of planned physical activities and integrating additional physical activity scores into a comprehensive physical activity score. This will provide a more accurate reflection of self-monitoring and, ideally, increase participants’ motivation [45].

The importance of SDM in the primary and secondary prevention of CVD is well recognized and reported in the literature, including in the context of physical activity and exercise prescription [46,47]. Overall, the focus on SDM in the intervention was well received by the HCPs, and they made appropriate efforts to integrate SDM into their interactions with participants. In addition to positive Observer Option^5^ ratings, the HCPs’ self-assessment of SDM (SDM-Q-Doc) showed continuous growth in their confidence with SDM skills. This indicates the need for thorough preparation and understanding of SDM among HCPs from an early start. An opportunity to improve this would be a (virtual) refresher workshop held shortly before the start of the study, along with a paper-based checklist to support them during the physical activity planning session with participants. These 2 strategies could enhance SDM skills from the beginning in the future RCT.

The HCPs and patients shared positive experiences of the study design. One of the HCPs suggested improvements to better coordinate tasks among multiple HCPs at the same study site to reduce misunderstandings. Some patients experienced the use of 2 wearable devices and the high number of questionnaires during the assessments as somewhat irritating; therefore, both should be reconsidered in a future RCT. Despite these manageable burdens, participants also expressed skepticism about the 6- to 12-month duration of a definitive RCT. The fast pace of digital technology development calls for efficient evaluation designs, so that potential patient benefits can be demonstrated quickly and DHIs may be implemented in clinical practice as soon as possible [32]. This needs to be balanced against the problem that the aktivplan DHI seeks to address, which is long-term maintenance of heart-healthy physical activity behavior and which requires follow-up periods of at least 6 months [48]. As both the study design and the aktivplan digital intervention will be refined based on this valuable pilot study dataset, design decisions need to be made to avoid discouraging potential participants during recruitment and to reduce the risk of attrition during a study with long-term follow-up.

Although this pilot study was not designed to provide generalizable effectiveness estimates, several insights may inform the design of similar DHIs in rehabilitation and post-rehabilitation settings. First, digital interventions should assume routine phone use but cannot rely on previous experience with activity trackers. Second, they should support flexible scheduling, integration of unplanned activities into feedback graphs, and a need for low-burden documentation (eg, automated tracking via wearables). Third, experiences of HCPs underline that even well-received digital tools require clear communication, timely feedback options, and pragmatic training and refresher structures to sustain implementation in routine care. Finally, time constraints appear to be the greatest barrier for HCPs to consistently apply SDM, at least in digital health contexts.

### Limitations

The context-specific development of the study intervention restricts the intervention’s transferability to other countries. Implementing aktivplan elsewhere would require assessing its compatibility with national regulatory frameworks, clinical pathways, and local sociocultural conditions [49]. Another limitation of the study is the lack of blinding. Blinding patients and HCPs delivering the intervention was not feasible. Although blinding outcome assessors would have been possible in theory, early discussions with the site principal investigators revealed that this was impractical due to limited staff and close working relationships at the participating sites. As a result, outcome assessments were conducted without blinding.

The study period was limited to 10 weeks, whereas a subsequent study would envisage a follow-up period of up to 1 year. This shorter duration was intentionally selected due to the exploratory nature of this pilot feasibility study. Finally, no predefined criteria were established to determine if and when to proceed to a full-scale trial [20]. As comparable Austrian data are unavailable and the funding organization does not specify progression criteria, decisions about a future definitive trial will be made collaboratively with key stakeholders, guided by the findings from this pilot study and evidence from comparable international research.

### Conclusions

Evidence-based interventions are needed to support regular physical activity after discharge from phase II rehabilitation for individuals with CVD or those at risk of CVD. DHIs hold promise to support these needs. This pilot feasibility study provided critical and detailed quantitative and qualitative information on recruitment rate, attrition, data completeness, adverse event reporting, patient adherence, intervention fidelity, and patients’ and HCPs’ experiences of the intervention and study procedures. These outcomes, in comparison with the existing literature, will guide the design of a future full-scale effectiveness trial of the aktivplan DHI. The study also identified areas for further refinement of the aktivplan app before conducting a definitive trial and offered useful insights for planning the future implementation of the intervention.

## Supplementary material

10.2196/90203 Multimedia Appendix 1 Interview schedule.

10.2196/90203 Multimedia Appendix 2 Focus group topic guide.

10.2196/90203 Checklist 1 TiDieR checklist.

10.2196/90203 Checklist 2 CONSORT 2010, checklist of information to include when reporting a pilot or feasibility trial.

## Abbreviations

CONSORT: Consolidated Standards of Reporting Trials
CVD: cardiovascular disease
DHI: digital health intervention
G-NoMAD: German Normalization Measure Development
HCP: health care professional
RCT: randomized controlled trial
SDM: shared decision-making
SDM-Q-Doc: Shared Decision Making Questionnaire
TIDieR: Template for Intervention Description and Replication

## References

[1] Roth GA, Mensah GA, Johnson CO (2020). Global burden of cardiovascular diseases and risk factors, 1990–2019. J Am Coll Cardiol.

[2] (2025). Jahrbuch Der Gesundheitsstatistik 2023.

[3] Niebauer J (2018). Cardiac rehabilitation in Austria. Wien Med Wochenschr.

[4] Schwaab B, Bjarnason-Wehrens B, Meng K (2021). Cardiac rehabilitation in German speaking countries of Europe-evidence-based guidelines from Germany, Austria and Switzerland LLKardReha-DACH-Part 2. J Clin Med.

[5] Ambrosetti M, Abreu A, Corrà U (2021). Secondary prevention through comprehensive cardiovascular rehabilitation: from knowledge to implementation. 2020 update. A position paper from the Secondary Prevention and Rehabilitation Section of the European Association of Preventive Cardiology. Eur J Prev Cardiol.

[6] ter Hoeve N, Huisstede BMA, Stam HJ, van Domburg RT, Sunamura M, van den Berg-Emons RJG (2015). Does cardiac rehabilitation after an acute cardiac syndrome lead to changes in physical activity habits? Systematic review. Phys Ther.

[7] (2019). Challenges and opportunities for cardiovascular disease research: Strategic Research Agenda for Cardiovascular Diseases (SRA-CVD). https://sku-l.de/wp-content/uploads/2019/05/Brosch_ERA-CVD_SRA_04-2019.pdf.

[8] Visseren FLJ, Mach F, Smulders YM (2021). 2021 ESC Guidelines on cardiovascular disease prevention in clinical practice. Eur Heart J.

[9] Wongvibulsin S, Habeos EE, Huynh PP (2021). Digital health interventions for cardiac rehabilitation: systematic literature review. J Med Internet Res.

[10] Luijk A, Mortensen SR, Hamborg TG (2024). The effectiveness of digital health interventions for the maintenance of physical activity following cardiac rehabilitation: a systematic review and meta-analysis. Digit Health.

[11] Meinhart F, Stütz T, Sareban M, Kulnik ST, Niebauer J (2020). Mobile technologies to promote physical activity during cardiac rehabilitation: a scoping review. Sensors (Basel).

[12] Bonneux C, Mahmood DY, Scherrenberg M (2022). The CoroPrevention-SDM Approach: A Technology-Supported Shared Decision Making Approach for A Comprehensive Secondary Prevention Program for Cardiac Patients.

[13] Gibson I, Jennings C, Neubeck L (2023). Using a digital health intervention “INTERCEPT” to improve secondary prevention in coronary heart disease (CHD) patients: protocol for a mixed methods non-randomised feasibility study. HRB Open Res.

[14] (2023). Classification of Digital Interventions, Services and Applications in Health: A Shared Language to Describe the Uses of Digital Technology for Health.

[15] Wurhofer D, Strumegger EM, Hussein R, Stainer-Hochgatterer A, Niebauer J, Kulnik ST (2022). The development of a digital tool for planning physical exercise training during cardiac rehabilitation. Stud Health Technol Inform.

[16] Marcos TA, Crutzen R, Leitner V (2024). Making it transparent: a worked example of articulating programme theory for a digital health application using Intervention Mapping. Digit Health.

[17] Cieza A, Kwamie A, Magaqa Q (2022). Framing rehabilitation through health policy and systems research: priorities for strengthening rehabilitation. Health Res Policy Syst.

[18] Lunz L, Würth S, Kulnik ST (2025). Health care professionals’ use of digital technology in the secondary prevention of cardiovascular disease in austria: online survey study. JMIR Cardio.

[19] Leysen D, Reich B, Carrozzo AE (2025). Feasibility of the aktivplan digital health intervention for regular physical activity following phase II rehabilitation: protocol for a mixed method randomized controlled pilot study (ACTIVE-CaRe Pilot). JMIR Res Protoc.

[20] Eldridge SM, Chan CL, Campbell MJ (2016). CONSORT 2010 statement: extension to randomised pilot and feasibility trials. BMJ.

[21] Hoffmann TC, Glasziou PP, Boutron I (2014). Better reporting of interventions: template for intervention description and replication (TIDieR) checklist and guide. BMJ.

[22] Wurhofer D, Neunteufel J, Strumegger EM (2023). Investigating shared decision-making during the use of a digital health tool for physical activity planning in cardiac rehabilitation. Front Digit Health.

[23] Medizinische Rehabilitation und Gesundheitsvorsorge. Pensionsversicheringsanstalt.

[24] Fachgesellschaften aus deutschland, österreich und der schweiz (d-ACH) S3-leitlinie zur kardiologischen rehabilitation (LL-kardreha) im deutschsprachigen raum europas deutschland, österreich, schweiz (d-a-CH). AWMF.

[25] Doherr H, Christalle E, Kriston L, Härter M, Scholl I (2017). Use of the 9-item Shared Decision Making Questionnaire (SDM-Q-9 and SDM-Q-Doc) in intervention studies-a systematic review. PLoS ONE.

[26] Barr PJ, O’Malley AJ, Tsulukidze M, Gionfriddo MR, Montori V, Elwyn G (2015). The psychometric properties of Observer OPTION(5), an observer measure of shared decision making. Patient Educ Couns.

[27] Rapley T, Girling M, Mair FS (2018). Improving the normalization of complex interventions: part 1 - development of the NoMAD instrument for assessing implementation work based on normalization process theory (NPT). BMC Med Res Methodol.

[28] Gale NK, Heath G, Cameron E, Rashid S, Redwood S (2013). Using the framework method for the analysis of qualitative data in multi-disciplinary health research. BMC Med Res Methodol.

[29] Kulnik ST, Gutenberg J, Mühlhauser K, Topolski T, Crutzen R (2023). Translation to German and linguistic validation of the Rapid Assessment of Physical Activity (RAPA) questionnaire. J Patient Rep Outcomes.

[30] Knight JF, Baber C (2005). A tool to assess the comfort of wearable computers. Hum Factors.

[31] (2025). Rehabilitationsplan 2025. https://www.sozialversicherung.at/cdscontent/load?contentid=10008.799150&version=1761222484.

[32] Pham Q, Wiljer D, Cafazzo JA (2016). Beyond the randomized controlled trial: a review of alternatives in mHealth clinical trial methods. JMIR Mhealth Uhealth.

[33] Taylor JL, Holland DJ, Keating SE (2020). Short-term and long-term feasibility, safety, and efficacy of high-intensity interval training in cardiac rehabilitation: the FITR Heart Study randomized clinical trial. JAMA Cardiol.

[34] Petersen AK, Oestergaard LG, van Tulder M, Laustsen S (2020). A comparison of high versus low dose of exercise training in exercise-based cardiac rehabilitation: a randomized controlled trial with 12-months follow-up. Clin Rehabil.

[35] Pinto BM, Goldstein MG, Papandonatos GD (2011). Maintenance of exercise after phase II cardiac rehabilitation. Am J Prev Med.

[36] Brouwers RWM, Kraal JJ, Regis M, Spee RF, Kemps HMC (2021). Effectiveness of cardiac telerehabilitation with relapse prevention. J Am Coll Cardiol.

[37] Meyerowitz-Katz G, Ravi S, Arnolda L, Feng X, Maberly G, Astell-Burt T (2020). Rates of attrition and dropout in app-based interventions for chronic disease: systematic review and meta-analysis. J Med Internet Res.

[38] Crutzen R, Viechtbauer W, Spigt M, Kotz D (2015). Differential attrition in health behaviour change trials: a systematic review and meta-analysis. Psychol Health.

[39] Marino M, Lucas J, Latour E, Heintzman JD (2021). Missing data in primary care research: importance, implications and approaches. Fam Pract.

[40] Kost RG, de Rosa JC (2018). Impact of survey length and compensation on validity, reliability, and sample characteristics for ultrashort-, short-, and long-research participant perception surveys. J Clin Transl Sci.

[41] Krosnick JA (1991). Response strategies for coping with the cognitive demands of attitude measures in surveys. Appl Cogn Psychol.

[42] Cohen Rodrigues TR, Reijnders T, de Buisonjé DR (2022). Lifestyle support preferences of patients with cardiovascular diseases: what lifestyle support might work best for whom?. PEC Innov.

[43] Anttila MR, Kivistö H, Piirainen A (2019). Cardiac rehabilitees’ technology experiences before remote rehabilitation: qualitative study using a grounded theory approach. J Med Internet Res.

[44] Laranjo L, Ding D, Heleno B (2021). Do smartphone applications and activity trackers increase physical activity in adults? Systematic review, meta-analysis and metaregression. Br J Sports Med.

[45] Bentlage E, Nyamadi JJ, Dubbeldam R (2023). The importance of activating factors in physical activity interventions for older adults using information and communication technologies: systematic review. JMIR Mhealth Uhealth.

[46] Turkson-Ocran RAN, Ogunwole SM, Hines AL, Peterson PN (2021). Shared decision making in cardiovascular patient care to address cardiovascular disease disparities. J Am Heart Assoc.

[47] Hansen D, Abreu A, Ambrosetti M (2022). Exercise intensity assessment and prescription in cardiovascular rehabilitation and beyond: why and how: a position statement from the Secondary Prevention and Rehabilitation Section of the European Association of Preventive Cardiology. Eur J Prev Cardiol.

[48] Gold N, Yau A, Rigby B, Dyke C, Remfry EA, Chadborn T (2021). Effectiveness of digital interventions for reducing behavioral risks of cardiovascular disease in nonclinical adult populations: systematic review of reviews. J Med Internet Res.

[49] Caiani EG, Kemps H, Hoogendoorn P (2024). Standardized assessment of evidence supporting the adoption of mobile health solutions: a clinical consensus statement of the ESC Regulatory Affairs Committee. European Heart Journal - Digital Health.

