# Supplementary material for: Feasibility of the Aktivplan Digital Health Intervention for Supporting Regular Physical Activity Following Phase II Rehabilitation: Randomized Controlled Pilot Feasibility Study (ACTIVE-CaRe Pilot)
Source: JMIR Form Res. 2026 Aug 11;10:e90203. doi: 10.2196/90203 (PMC13482960; doi:10.2196/90203)
Supplement: Supplementary file 1 [file formative-v10-e90203-s001.pdf]

Online supplement 3. Qualitative interview schedule (authors' translation from the original German to English).

Qualitative interview - control group / week 10 (final study visit)

Date: |____|___|/|___|___|/|___|___| Initials: ☐
Carried out? ☐ Yes ☐ No
Reason if not: ______________________________

Checklist:
- ☐ Tape recording started
- ☐ Participant information sheet at hand
- ☐ Interviewer speaks on tape: *ACTIVE-CaRe Pilot Interview on <date> at <time> with study participant <participant ID>*

Process evaluation

- How did you feel about being invited to take part in the study?
  What motivated you to take part in this study?
- What did you think of the participant information sheet? *(Note: show PIS to participant)*
- What did you think of the conversation(s) to inform you about the study?
  Were all your questions sufficiently answered before you decided to participate?

- How did you feel when you were told that you were assigned to the control group?
- What do you think of the opportunity to use the app after you complete your study participation?

- From your point of view, how could such an offer be designed if the study is conducted for a longer period (6-12 months)?
- How did you find the additional study examinations and questionnaires?
- Do you have any suggestions on how we can improve the study process to make it more pleasant and uncomplicated for patients?

- Have you organised support for regular exercise on your own initiative since you were discharged from rehabilitation? (**aktiv**plan excluded / other apps / sports groups / personal trainer / physiotherapy, etc.)
  If "Yes", what did you organise and how much did you spend on it?

- Finally, is there anything else you would like to tell us / that has not yet been discussed?

Qualitative interview - intervention group / week 10 (final study visit)

Date: |____|__|/|__|__|/|__|__|

Carried out? ☐ Yes ☐ No

Reason if not:

Initials: ☐

Checklist:

☐ Tape recording started

☐ Participant information sheet at hand

☐ Interviewer speaks on tape: *ACTIVE-CaRe Pilot Interview on <date> at <time> with study participant <participant ID>*

Process evaluation

- How did you feel about being invited to take part in the study?
  What motivated you to take part in this study?
- What did you think of the participant information sheet? *(Note: show PIS to participant)*
- What did you think of the conversation(s) to inform you about the study?
  Were all your questions sufficiently answered before you decided to participate?

- Was the offer to be able to use the **aktiv**plan app after completing your study participation, even if you had been assigned to the control group, something that influenced your decision to participate?
- How could such an offer be designed if the study is conducted for a longer period (6-12 months)?
- How did you find the additional study examinations and questionnaires?
- Do you have any suggestions on how we can improve the study process to make it more pleasant and uncomplicated for patients?

- In addition to **aktiv**plan, have you organised support for regular exercise on your own initiative since you were discharged from rehabilitation? (**aktiv**plan excluded / other apps / sports groups / personal trainer / physiotherapy, etc.)
  If "Yes", what did you organise and how much did you spend on it?

Questions on intervention

- How did you experience the physical activity planning session (during which your physical activity plan was drawn up)?
- Your experience with the **aktiv**plan app over the past few weeks is very valuable for the further development of the app. Is there anything you would like to pass on to the researchers and developers of the app?
- In this study, you used the **aktiv**plan app for 10 weeks. Imagine you were to use the app for a longer period, e.g. 6-12 months. How would you find that?
- Were there any difficulties or challenges (in the context of the study / specifically in relation to **aktiv**plan) for you?
- Finally, is there anything else you would like to tell us / that has not yet been discussed?
