# Supplementary material for: Feasibility of the Aktivplan Digital Health Intervention for Supporting Regular Physical Activity Following Phase II Rehabilitation: Randomized Controlled Pilot Feasibility Study (ACTIVE-CaRe Pilot)
Source: JMIR Form Res. 2026 Aug 11;10:e90203. doi: 10.2196/90203 (PMC13482960; doi:10.2196/90203)
Supplement: Supplementary file 3 [file formative-v10-e90203-s003.pdf]

Online supplement 3. Description of the **aktiv**plan digital health intervention according to the TIDieR framework (1).

Item number	Item
1	*BRIEF NAME – Provide the name or a phrase that describes the intervention.*
	**aktiv**plan<br><br>The **aktiv**plan digital health intervention consists of a digital planning calendar for regular heart-healthy exercise. Healthcare professionals have access to this planning calendar via a website (view for healthcare professionals). Patients have access to the planning calendar via an app on their smartphone or tablet or, if required, via a website on a smartphone, tablet or computer (view for patients).<br><br>The planning calendar is introduced to the patient by a healthcare professional towards the end of rehabilitation phase II during a physical activity planning session. Together, the healthcare professional and patient enter a personalised physical activity plan in accordance with the patient's rehabilitation programme into the planning calendar. This physical activity planning session is conducted by the healthcare professional according to the principles of shared decision-making. A personalised heart-healthy physical activity plan for the patient is jointly agreed, which the patient should carry out independently after completing the rehabilitation phase II. This agreed physical activity plan is entered into the planning calendar by the healthcare professional via the **aktiv**plan website (view for healthcare professionals).<br><br>The digital planning calendar can be viewed and edited by the patient via the **aktiv**plan app on a smartphone or tablet or, if required, via a website on a computer. The patient can mark planned physical activity sessions as completed or enter additional unplanned sessions ("extra activities"). The patient can call up short videos with exercise instructions via the **aktiv**plan app and export a documentation sheet on which all the activities performed are listed. This documentation sheet can be used for the patient's own review and motivation, or can be passed on to the healthcare professional for review and documentation.<br><br>On the **aktiv**plan website (view for healthcare professionals), the healthcare professional can view the planning calendars of all patients under their care and see the adherence of all patients in an overview table ("traffic light display"). Other functions of the **aktiv**plan intervention include automated messages to the patient with motivational content or reminders for planned physical activity sessions, personalised goal-setting and the option for the healthcare professional to give the patient feedback and contact them via the app using a written message (in-app message, push notification). At any follow-up appointments with the healthcare professional, the patient can reflect on the previous physical activity plan and discuss the further plan.<br><br>A brief video describing the **aktiv**plan intervention is available here: https://dhp.lbg.ac.at/wp-content/uploads/sites/8/2021/12/aktivplan_v4.mp4
2	*WHY – Describe any rationale, theory, or goal of the elements essential to the intervention.*
	The problem of long-term habit formation and maintenance of regular heart-healthy physical activity after completion of phase II cardiac rehabilitation is well-described in the scientific literature. The scientific literature also points to a possible solution through digital technologies and digital health interventions.<br><br>In Austria, there is an urgent need to provide patients with such an offer to digitally support long-term habit formation and the maintenance of regular heart-healthy physical activity. This offer should be made available to patients with cardiovascular disease after completion of

	cardiac rehabilitation phase II, as well as to patients who participate in rehabilitation phase II due to non-cardiac indications and who have cardiovascular risk factors.<br><br>In **aktiv**plan, several scientifically underpinned psychological approaches to healthy behaviour change are taken into account and implemented: planned behaviour, self-efficacy, personalised goal-setting, monitoring and feedback, as well as the recognised authority of the health expert (2).
3	*WHAT – Materials: Describe any physical or informational materials used in the intervention, including those provided to participants or used in intervention delivery or training of intervention providers. Provide information on where the materials can be accessed (e.g., online appendix, URL).*
	The patient requires a smartphone compatible with the **aktiv**plan app (operating system Android 4.4 / Apple iOS 11.0 or higher) and with internet access. The patient should generally have a good internet connection on their smartphone at home and in their neighbourhood. The **aktiv**plan app is listed in the Google Play Store and in the Apple App Store. The **aktiv**plan app is downloaded by the patient in the presence and under the guidance of the healthcare professional and set up on the patient's smartphone. The patient requires an email account (email address) to receive a personal link from the healthcare professional, allowing the patient to log in to the **aktiv**plan app.<br><br>The healthcare professional requires a computer to open the view for healthcare professionals on the **aktiv**plan website. The computer should be set up in a room where the physical activity planning session can be conducted in a quiet and relaxed atmosphere. The computer screen should be large enough and positioned in such a way that both the healthcare professional and the patient can comfortably follow the actions on the screen at the same time.
4	*WHAT – Procedures: Describe each of the procedures, activities, and/or processes used in the intervention, including any enabling or supporting activities.*
	The following activities and processes take place when using **aktiv**plan:<br><br>A. In the last week of rehabilitation phase II, a one-hour physical activity planning session takes place during which the patient installs the **aktiv**plan app on their own smartphone or tablet. The healthcare professional opens the **aktiv**plan website (view for healthcare professionals) on the computer. A heart-healthy physical activity plan is agreed and entered into the planning calendar by the healthcare professional. The patient formulates personal physical activity goals, which are also entered into the planning calendar by the healthcare professional.<br><br>B. The patient has access to the agreed physical activity plan via the **aktiv**plan app (view for patients). After discharge from rehabilitation phase II, the patient carries out the agreed physical activity plan independently and checks off all completed activities in the planning calendar. The patient receives regular automated reminder messages (in-app and push notifications), and messages with motivational content and helpful tips. Via the **aktiv**plan app, the patient can access videos with exercise instructions.<br><br>C. After the patient's discharge from rehabilitation phase II, the healthcare professional monitors the patient's adherence once a week using the **aktiv**plan patient overview (view for healthcare professionals). The "traffic light display" indicates how many of the planned physical activity sessions have been carried out by the patients over the past 4 weeks (green ≥80%, yellow 50-79%, red <50% of the planned physical activity minutes). If the indicator is red, the healthcare professional initiates a message to the patient to enquire about the reasons for the low adherence and possible need for further discussion. If necessary, further discussion is offered, which can be conducted in writing (email), by telephone, video call or in person, depending on the preference of the healthcare professional and the patient. The patient can indicate a change in their status in the app, including *on holiday*,

	unwell, and *physical activity plan unsuitable.* This status is visible to the healthcare professional and can be used to explain low adherence. When the patient changes their status to *physical activity plan unsuitable*, the healthcare professional also initiates a message to the patient and offers further discussion and review of the physical activity plan.<br><br>Please refer to (2) for screenshots of the **aktiv**plan application.<br><br>Classified according to the World Health Organisation's Classification of digital interventions, services and applications in health (3), the **aktiv**plan digital health intervention incorporates the following functions:<br>1.1.2 Transmit targeted health information to person(s) based on health status or demographics;<br>1.1.3 Transmit targeted alerts and reminders to person(s);<br>1.4.2 Self-monitoring of health or diagnostic data by the individual;<br>1.4.3 Active data capture/documentation by an individual;<br>1.6.2 Simulated human-like conversations with individual(s);<br>1.8.1 Manage provision and withdrawal of consent by individuals;<br>2.1.1 Verify a person's unique identity;<br>2.1.2 Enroll person(s) for health services/clinical care plan;<br>2.2.1 Longitudinal tracking of person's health status and services;<br>2.2.4 Routine health indicator data collection and management;<br>2.3.2 Provide checklist according to protocol;<br>2.4.2 Remote monitoring of person's health or diagnostic data by provider;<br>2.5.4 Transmit non-routine health event alerts to healthcare provider(s); and<br>2.7.1 Identify persons in need of services (3).
5	*WHO PROVIDED – For each category of intervention provider (e.g. psychologist, nursing assistant), describe their expertise, background and any specific training given.*
	The **aktiv**plan digital health intervention (setting up the **aktiv**plan app together with the patient, individual physical activity planning session with personalised physical activity planning, tracking the patient's adherence via the **aktiv**plan website) is carried out by healthcare professionals (rehabilitation staff). Healthcare professionals should have relevant professional qualifications (e.g., sports science, physiotherapy, exercise physiology) and sound previous experience and expertise in personalised physical activity planning and exercise prescription for cardiac patients (secondary prevention) and for patients with increased cardiovascular risk (primary prevention).<br><br>The rehabilitation staff carrying out the **aktiv**plan digital health intervention receive an introduction to **aktiv**plan in the form of a one-day workshop organised by the developers of **aktiv**plan. This introduction includes:<br>• Presentations on the development process and the scientific background of the **aktiv**plan digital health intervention<br>• Demonstration of the **aktiv**plan application<br>• Practical exercise of the physical activity planning session using **aktiv**plan ("role play")<br>• Guidance on trouble-shooting technical issues with **aktiv**plan<br>• Self-directed practice of the **aktiv**plan functions<br>• Opportunity for questions and discussion with the developers of **aktiv**plan

6	*HOW – Describe the modes of delivery (e.g. face-to-face or by some other mechanism, such as internet or telephone) of the intervention and whether it was provided individually or in a group.*
	The physical activity plan is drawn up in a one-to-one conversation between the healthcare professional and the patient according to the principles of shared decision-making (4). The patient is responsible for carrying out the planned physical activity sessions and can enter new (unplanned) sessions. The patient should check off all completed physical activity sessions (planned and unplanned) in the **aktiv**plan app. The patient receives automated messages to their **aktiv**plan app (in-app and push notifications), which contain motivational content or reminders of planned physical activity sessions and personal goals.<br><br>The healthcare professional monitors the patient's adherence in the **aktiv**plan application. It is possible for the healthcare professional to give the patient feedback via the app and make contact (personalised in-app and push notification). It is possible for the patient to communicate a change in status to the healthcare professional via the app (e.g., *on holiday*, *unwell*, or *physical activity plan unsuitable*).
7	*WHERE – Describe the type(s) of location(s) where the intervention occurred, including any necessary infrastructure or relevant features.*
	The **aktiv**plan digital health intervention is initiated at the rehabilitation centre (physical activity planning session between patient and healthcare professional). A room is required that allows for a one-hour conversation in a quiet and relaxed atmosphere, and in which a computer with internet access is available. The computer screen should be large enough and positioned in such a way that both the healthcare professional and the patient can comfortably follow what is happening on the screen in real time. The room should allow for good mobile phone reception, as the patient should be able to receive an email and download the **aktiv**plan app on their smartphone during the session.
8	*WHEN and HOW MUCH – Describe the number of times the intervention was delivered and over what period of time including the number of sessions, their schedule, and their duration, intensity or dose.*
	The physical activity planning session takes place once in rehabilitation phase II. Sixty minutes should be scheduled for the physical activity planning session between the patient and the healthcare professional. In addition, the time required to prepare for and follow up on the session should be planned (e.g., for reserving the room, scheduling the appointment for the patient, possibly accompanying the patient to/from the room, and documenting the session in the patient's medical notes).<br><br>After this, the patient uses the **aktiv**plan app independently, regularly checking the physical activity plan, checking off the planned activities that have been carried out, and entering additional unplanned activities. The time required depends on the frequency of the planned activities. For example, with three planned activities per week and two additional unplanned activities per week, the weekly time required is approx. 25-50 minutes (approx. 5-10 minutes per activity). In the ACTIVE-CaRe Pilot study, the patient uses the **aktiv**plan app for 10 weeks following discharge from rehabilitation phase II.<br><br>During patients' independent use of the **aktiv**plan app, healthcare professionals (rehabilitation staff) track the adherence of all patients currently using **aktiv**plan on a weekly basis. At <50% (red indicator), the healthcare professional initiates a message to the patient to enquire about reasons for the low adherence and possible need for further discussion. As part of the ACTIVE-CaRe Pilot study, the app is used by a maximum of 20 patients per rehabilitation centre at any one time. Depending on the proportion of patients with adherence <50%, the weekly time required for healthcare professionals is estimated at between 10 and 60 minutes.

9	*TAILORING – If the intervention was planned to be personalised, titrated or adapted, then describe what, why, when, and how.*
	As part of the **aktiv**plan digital health intervention, a one-to-one physical activity planning session takes place between the patient and the healthcare professional. This session is conducted by the healthcare professional according to the principles of shared decision-making (4) in order to jointly agree on a heart-healthy physical activity plan that is personalised to the patient's preferences and capablities.<br><br>The physical activity plan is personalised taking into account the patient's physiological parameters relevant to exercise training (e.g., maximum heart rate and exercise capacity) as well as the patient's personal preferences and wishes with regard to types of sport / types of physical activity and compatibility with the patient's everyday life.
10*	*MODIFICATIONS – If the intervention was modified during the course of the study, describe the changes (what, why, when, and how).*
	N/A
11	*HOW WELL – Planned: If intervention adherence or fidelity was assessed, describe how and by whom, and if any strategies were used to maintain or improve fidelity, describe them.*
	Für das Beratungsgespräch zwischen Patient und Gesundheitsexperten (Rehabilitationspersonal) beinhaltet die **aktiv**plan Webseite für Gesundheitsexperten einen Leitfaden für die partizipative Gesprächsführung (Shared Decision-Making) und eine Checkliste zur Erinnerung, auf welcher wesentliche Aspekte der partizipativen Gesprächsführung vom Gesundheitsexperten abgehakt werden können. Diese Maßnahme soll die Adhärenz der Gesundheitsexperten mit den Prinzipien der partizipativen Gesprächsführung unterstützen. Im Rahmen der ACTIVE-CaRe Pilot Studie erfolgt die Videoaufzeichnung der Beratungsgespräche und Analyse, um die Adhärenz der Gesundheitsexperten mit der partizipativen Gesprächsführung zu evaluieren.<br><br>Die Adhärenz der Patienten mit dem vereinbarten regelmäßigen Trainingsplan wird durch Selbsteingabe aller absolvierten Trainingsaktivitäten und der erreichten Ziele in der **aktiv**plan App erfasst und durch automatisierte motivierende Nachrichten und Erinnerungsnachrichten an die Patienten unterstützt. Daten zur Adhärenz der Patienten mit der Nutzung der **aktiv**plan App wird durch eine automatisierte Nutzungserfassung erhoben.<br><br>Die wöchentliche Übersicht der Adhärenz aller Patienten durch den Gesundheitsexperten und daraus erfolgte Kommunikation der Gesundheitsexperten mit Patienten werden vom Gesundheitsexperten dokumentiert.<br><br>For the physical activity planning session between patient and healthcare professional (rehabilitation staff), the **aktiv**plan website for healthcare professionals contains a guide to participatory dialogue (shared decision-making) and a checklist as a reminder, on which key aspects of participatory dialogue can be ticked off by the healthcare professional. This is intended to support the adherence of healthcare professionals to the principles of participatory dialogue and shared decision-making (4) during the physical activity planning session. In the ACTIVE-CaRe Pilot study, the physical activity planning session are video-recorded and analysed using the Observer OPTION 5 instrument (5) in order to evaluate the adherence of the healthcare professionals to the participatory dialogue and shared decision-making.<br><br>The adherence of patients to the agreed regular physical activity plan is recorded by self-entry of all completed physical activity sessions and personal goals in the **aktiv**plan app and supported by automated motivational messages and reminders to patients. Data on patients' adherence to using the **aktiv**plan app is collected through automated usage logging.

	The weekly review of the adherence of all patients by the healthcare professional and the resulting communication between the healthcare professional and patients are documented by the healthcare professional.
12*	*HOW WELL – Actual: If intervention adherence or fidelity was assessed, describe the*
	N/A

* If completing the TIDieR checklist for a protocol, these items are not relevant to the protocol and cannot be described until the study is complete.

References

1. Hoffmann TC, Glasziou PP, Boutron I, Milne R, Perera R, Moher D, et al. Better reporting of interventions: template for intervention description and replication (TIDieR) checklist and guide. BMJ. 2014 Mar 7;348:g1687.
2. Marcos TA, Crutzen R, Leitner V, Smeddinck JD, Strumegger EM, Wurhofer D, et al. Making it transparent: a worked example of articulating programme theory for a digital health application using Intervention Mapping. Digit Health. in press.
3. Classification of digital interventions, services and applications in health: a shared language to describe the uses of digital technology for health, 2nd ed [Internet]. Geneva; 2023 [cited 2024 May 2]. Available from: https://www.who.int/publications-detail-redirect/9789240081949
4. Wurhofer D, Neunteufel J, Strumegger EM, Höppchen I, Mayr B, Egger A, et al. Investigating shared decision-making during the use of a digital health tool for physical activity planning in cardiac rehabilitation. Front Digit Health. 2023;5:1324488.
5. Barr PJ, O'Malley AJ, Tsulukidze M, Gionfriddo MR, Montori V, Elwyn G. The psychometric properties of Observer OPTION(5), an observer measure of shared decision making. Patient Educ Couns. 2015 Aug;98(8):970–6.
